# Recurrent fusion RNA *DUS4L-BCAP29* in non-cancer human tissues and cells

**DOI:** 10.18632/oncotarget.16329

**Published:** 2017-03-17

**Authors:** Yue Tang, Fujun Qin, Aiqun Liu, Hui Li

**Affiliations:** ^1^ College of Life Sciences, Zhengzhou University, Zhengzhou, Henan 450008, P.R. China; ^2^ Department of Pathology, School of Medicine, University of Virginia, Charlottesville, VA 22908, USA; ^3^ College of Basic Medical Sciences, Zhengzhou University, Zhengzhou, Henan, 450001, P.R. China; ^4^ Department of Endoscopy, The Affiliated Tumor Hospital of Guangxi Medical University, Nanning, Guangxi 530021, P.R. China

**Keywords:** DUS4L-BCAP29, chimeric RNA, cis-SAGe, fusion transcript

## Abstract

Traditional gene fusions are involved in the development of various neoplasia. *DUS4L-BCAP29*, a chimeric fusion RNA, has been reported to be a cancer-fusion in prostate and gastric cancer, in addition to playing a tumorigenic role. Here, we showed that the *DUS4L-BCAP29* fusion transcript exists in a variety of normal tissues. It is also present in non-cancer epithelial, as well as in fibroblast cell lines. Quantitatively, the fusion transcript has a comparable expression in non-cancerous, gastric and prostate cell lines and tissues as in the cancer cell lines and tissues. The loss-of-function approach as previously reported is not sufficient to prove the functionality of the fusion. On the other hand, the gain-of-function approach showed that overexpression of *DUS4L-BCAP29* promotes cell growth and motility, even in non-cancer cells. Finally, we provide further evidence that the fusion transcript is a product of cis-splicing between adjacent genes. In summary, we believe that in contrast to traditional gene fusions, *DUS4L-BCAP29* cannot be used as a cancer biomarker. Instead, it is a fusion transcript that exists in normal physiology and that its pro-growth effect is not unique to cancer cells.

## INTRODUCTION

Chromosomal translocations that lead to gene fusions are well-known cancer-causing genetic events that are actively used in clinical cancer diagnosis, and their gene products have been shown to be effective targets for directed therapy [1–3]. Prominent examples include *BCR-ABL* in chronic myelogenous leukemia [4] with the development of Gleevec as a paradigm for targeted therapy [5], frequent gene fusion *TMPRSS2-ERG* in prostate cancer [6], and the rapid targeting of ALK gene fusion products with crizotinib after the discovery of *EML4-ALK* in lung cancer [7, 8]. The success of these discoveries has led to the prevailing view that gene fusions and fusion products (RNA and protein) are generated due to chromosomal rearrangement at the DNA level, and thus are unique to cancer. However, others and we have shown that fusion transcripts can also be detected in normal human cell lines [9] and tissues [10–17]. They may be products of intergenic splicing instead of traditional chromosomal rearrangement [18–20]. In a recent study, we found a large number of fusion RNAs by analyzing nearly 300 RNA-Seq libraries, covering 30 different non-neoplastic human tissues and cells [17]. From the study, we identified 291 recurrent fusions, 51 in more than five tissue types. Among them, *DUS4L-BCAP29*, was found in six different tissues.

The *DUS4L-BCAP29* fusion was previously discovered in gastric and prostate cancers [21, 22], and has been deposited in the Mitelman Database of Chromosome Aberrations and Gene Fusions in Cancer in the Cancer Genome Anatomy Project as a cancer-fusion. It was also reported to play a cancer-promoting role in gastric cancer [22], and proposed to be used as a cancer biomarker. However, our results showing its presence in multiple non-neoplastic tissues and cells, raise questions about its biomarker potential and challenge its relevance in tumorigenesis.

## RESULTS

### *DUS4L-BCAP29* is widely expressed in non-neoplastic human tissues and cell lines

**Figure 1:**
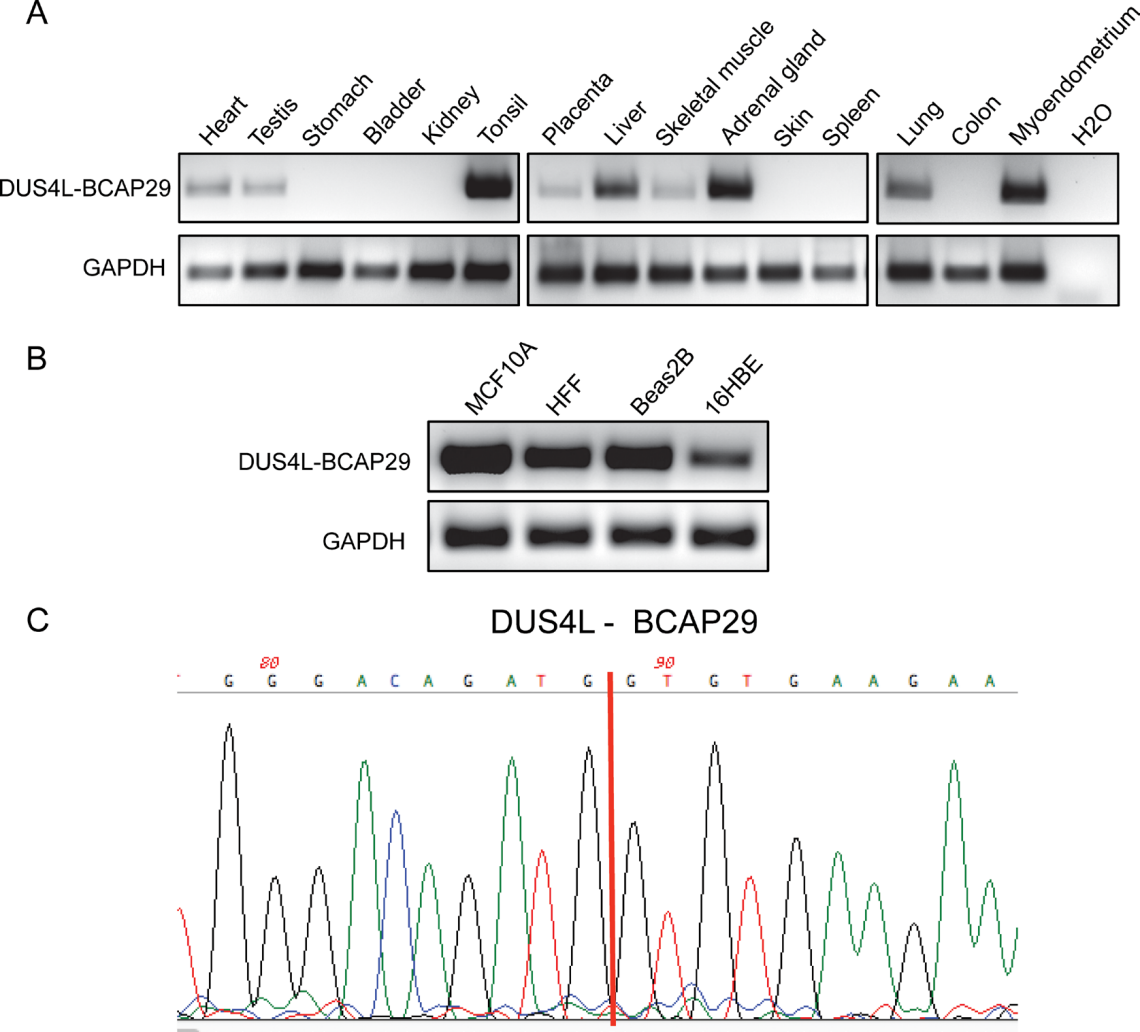
Detection of *DUS4L-BCAP29* in non-cancer human tissues and cell lines (**A**) Detection of the *DUS4L-BCAP29* in several non-cancer human tissues by RT-PCR and followed by agarose electrophoresis. *GAPDH* was used as internal control. (**B**) Detection of the *DUS4L-BCAP29* in several non-cancer cell lines by RT-PCR and followed by agarose electrophoresis. *GAPDH* was used as internal control. (**C**) Sanger sequencing validation of the RT-PCR product. Red line marks the fusion junction site.

*DUS4L-BCAP29* has been reported in both gastric and prostate cancers [21, 22]. However, recently, by analyzing RNA-Seq datasets, we detected the *DUS4L-BCAP29* fusion transcript in multiple non-neoplastic tissues [17]. To confirm the finding that the fusion transcript exists in non-cancerous tissues, we designed primers flanking the fusion junction site, and used RT-PCR to detect the fusion in our collection of normal tissues. As shown in Figure 1A, the fusion RNA was indeed detected in multiple tissues, ranging from heart to testis (Figure 1A). Furthermore, it is expressed in diverse non-cancerous cell lines, including mammary gland (MCF10A), lung epithelial (Beas2B and 16HBE), and foreskin fibroblast (HFF) (Figure 1B). It is the same fusion form, involving the first seven exons of the *DUS4L* gene, and the last seven exons of *BCAP29* as previously reported [22] (Supplementary Figure 1). The junction sequence is also identical to that reported in the gastric cancer study [22] (Figure 1C).

### *DUS4L-BCAP29* is not significantly overexpressed in gastric or prostate cancer cells

**Figure 2:**
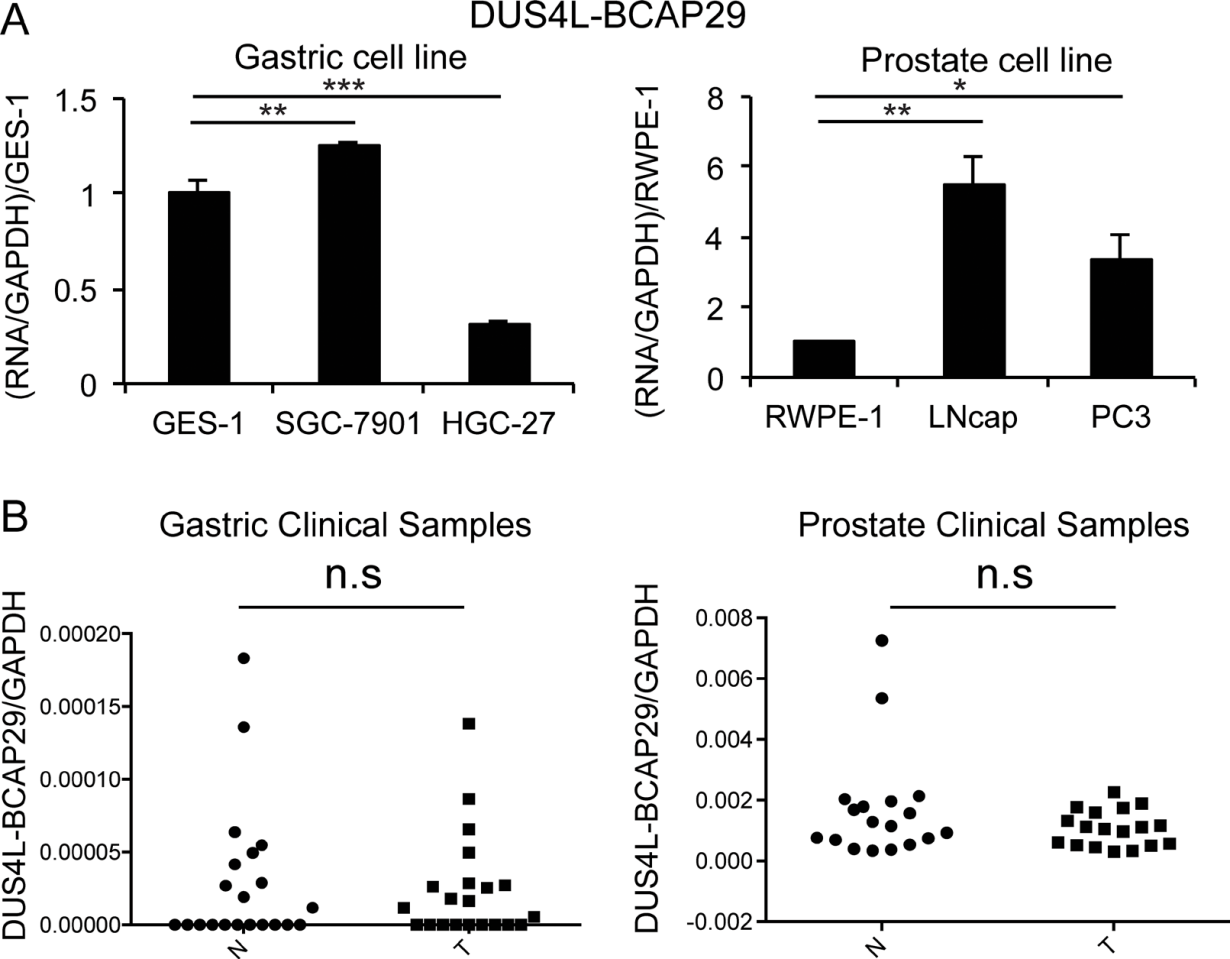
Quantification of *DUS4L-BCAP29* expression in gastric and prostate tissues and cell lines (**A**) qRT-PCR measuring *DUS4L-BCAP29* expression in cell lines. Left panel is the comparison in gastric cell lines, GES-1 and SGC-7901, HGC-27 cell lines. The expression was normalized to *GAPDH*, and then normalized to that in GES-1. Right panel is the comparison in prostate cell lines, RWPE-1, LNCaP, and, PC3 cell lines. The expression level was normalized to *GAPDH*, and then normalized to that in RWPE-1. (**B**) qRT-PCR measuring *DUS4L-BCAP29* expression in clinical samples. Left panel is the comparison in 21 gastric cancer and matched normals. Right panel is the comparison in 18 prostate cancer and 18 non-cancer prostate tissue samples. The expression level was normalized to *GAPDH*.

To test whether the fusion RNA is expressed at a much higher level in cancer vs. non-cancer cell lines, we used qRT-PCR to quantify the difference of expression in several gastric and prostate non-cancer (GES-1 and RWPE-1), and cancer lines (SGC-7901, HGC-27, LNCaP, and PC3). In the gastric cells, contradictory to the previous report [22], *DUS4L-BCAP29* is expressed at a comparable level in GES-1 as in SGC-7901, and even lower in HGC-27 (Figure 2A). In the prostate cells, the fusion is indeed expressed at lower levels in RWPE-1, than in LNCaP and PC3 cells (Figure 2A). We then compared the fusion RNA expression in clinical samples. No statistical difference was observed between the 21 gastric prostate cancer and normal matched pairs (Figure 2B). Similarly, no statistical difference was seen in 18 prostate cancer and 18 non-cancer prostate tissue samples (Figure 2B).

The fact that the fusion RNA is present in non-neoplastic tissues and cells, and expressed to a similar level raised the question of whether or not it is truly oncogenic, as previously suggested [22].

### Loss-of-function system

**Figure 3:**
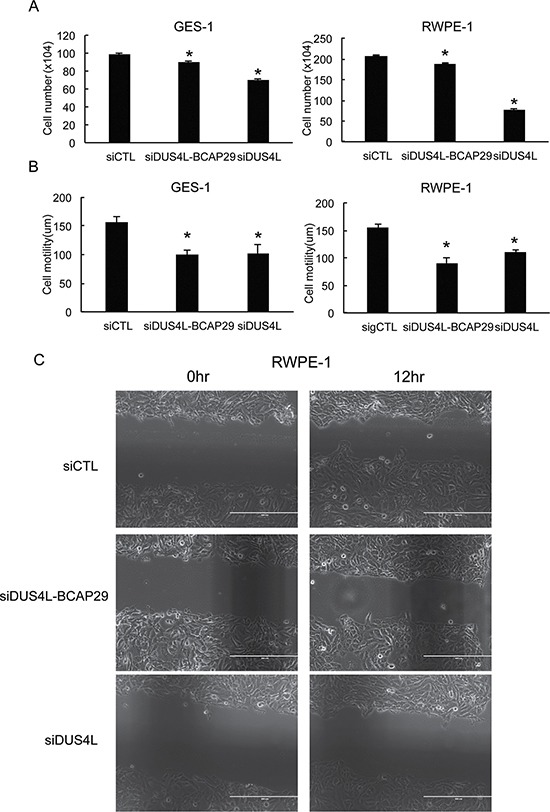
Loss-of-function system (**A**) siRNAs silencing *DUS4L-BCAP29* and wild type *DUS4L* decreased cell proliferation in GES-1 and RWPE-1 cell lines. The numbers of viable cells were counted three days after siRNA transfection. (**B**) siRNAs silencing *DUS4L-BCAP29* and wild type *DUS4L* decreased cell motility in GES-1 and RWPE-1 cell lines. Wound healing assay was conducted after the cells achieved monolayer confluency. Distance of the wound was measured right after scratch and at a later time point. Differences of the distance were plotted. *p < 0.05 (**C**) Representative wound-healing assay pictures showing reduced motility in siDUS4L-BCAP29 and siDUS4L transfected RWPE-1 cells, compared with cells transfected with control siCTL. The distances of the gaps were measured and differences between two time points were compared.

Previously, a siRNA (siDUS4L-BCAP29) targeting the fusion was used to demonstrate its role in gastric cancer [22]. However, the siRNA targeting sequence lies in the common region of the fusion and wild type *DUS4L* (Supplementary Figures 1 and 2), thus difficult to justify its specificity on the fusion transcript as reported [22]. Indeed, we found that the siRNA silences both the fusion (Supplementary Figure 3A) and the *DUS4L* wild transcripts (Supplementary Figure 3B). To silence the fusion transcript specifically, we usually design siRNAs targeting the junction sequence. In the past, we were able to use this approach to achieve fusion-specific silencing, including *SLC45A3-ELK4* [9, 23], *CTBS-GNG5*, and *CTNNBIP1-CLSTN1* [17]. Unfortunately, we failed to develop such a siRNA for *DUS4L-BCAP29*. To determine whether the previously reported reduced cell proliferation was truly due to the fusion RNA silencing, we designed another siRNA targeting only the wild type *DUS4L* (Supplementary Figures 1 and 2). Since the fusion is expressed in non-neoplastic tissues and cells, we tested the loss-of-function system in GES-1 and RWPE-1 cells. As expected, siDUS4L-BCAP29, but not siDUS4L silenced the fusion transcripts in both cell lines (Supplementary Figure 3A), whereas both siRNAs silenced the wild type transcript to a similar extent (Supplementary Figure 3A and 3B). When we measured cell proliferation, we found a more dramatic effect in siDUS4L-transfected cells in both GES-1, and RWPE-1 (Figure 3A). Both cell lines also showed significantly reduced migration ability when transfected with either siRNA, and to a similar extent (Figure 3B and 3C). We thus suspect that the effect of this loss-of-function system is mainly due to the silencing of wild type *DUS4L*. In conjunction with the previous report [22], we could not detect wild type *BCAP29*. Therefore, the effect of the siRNAs on *BCAP29* was not evaluated.

We then performed microarray analyses on the GES-1 samples transfected with the control siRNA, or siDUS4L-BCAP29, or siDUS4L. Consistent with the above observation, we noticed that the most significantly enriched Gene Ontology (GO) term is negative regulators of apoptosis, and this term is shared by both siDUS4L-BCAP29, and siDUS4L (Supplementary Figure 5). Interestingly, a few GO terms unique to the siDUS4L-BCAP29 were enriched, suggesting that the fusion may have some unique functions.

### Gain-of-function system

**Figure 4:**
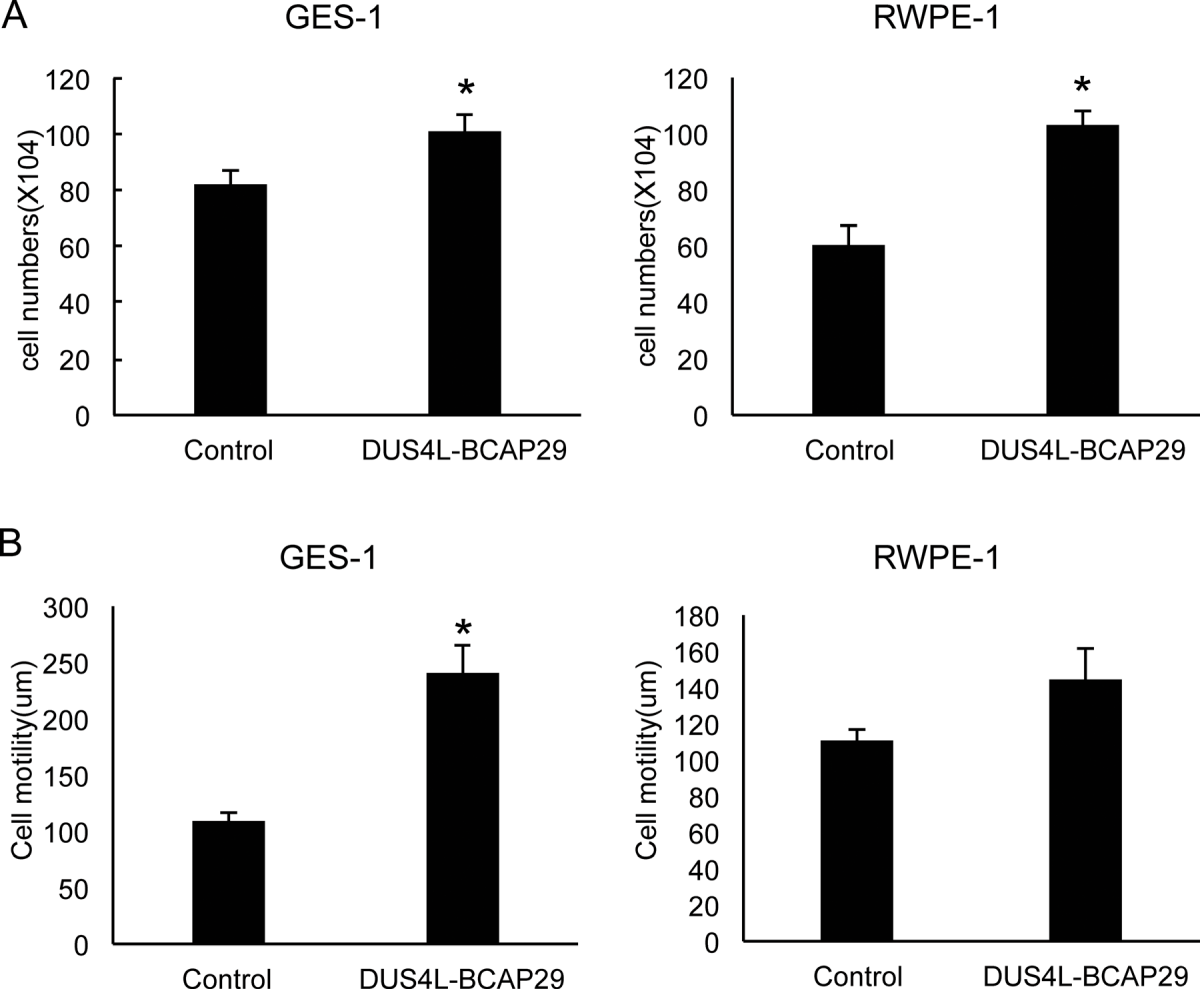
Gain-of-function system Cells were transfected with either empty vector “control” or the plasmid expressing the fusion “DUS4L-BCAP29”. Stable cells expressing the fusion were selected with corresponding antibiotics. (**A**) Overexpression of *DUS4L-BCAP29* promoted cell proliferation in GES-1 and RWPE-1 cell lines. (**B**) Overexpression of *DUS4L-BCAP29* promoted cell motility in GES-1 and RWPE-1 cell lines. Wound healing assays were conducted on stable cells as described before. *p < 0.05

Previously, a DUS4L-BCAP29 expression vector was transfected into gastric cancer cells to demonstrate its effect on cancer cell proliferation [22]. We decided to test the effect of fusion overexpression on non-cancer cells. The full length of *DUS4L-BCAP29* was cloned into a mammalian expression plasmid, pCDNA3.1, and a retroviral vector of pQXCIH-CMV. GES-1 and RWPE-1 cells were transfected, and stable cells were then selected by G418 (for pCDNA3.1), and hygromycin (for pQXCIH-CMV) respectively. In both systems, we observed enhanced proliferation rates in both cell lines (Figure 4A), and significantly increased cell motility, at least in GES-1 cells (Figure 4B). These results argue that the increased proliferation and motility effects of the fusion are not specific to cancer cells.

### *DUS4L-BCAP29* as a cis-SAGe fusion

**Figure 5:**
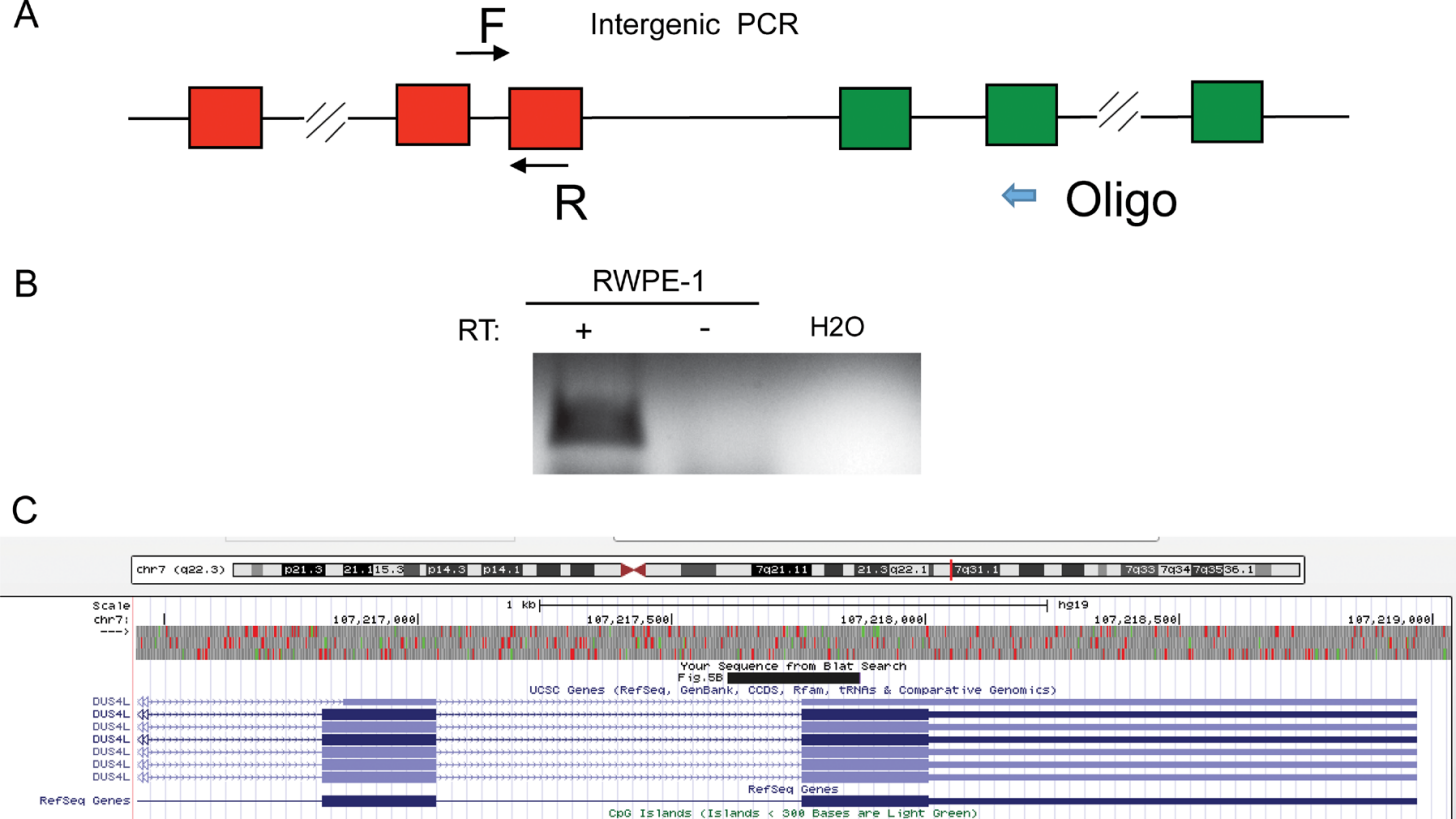
*DUS4L-BCAP29* is a product of transcriptional read-through (**A**) Scheme of the experiment. Reverse transcription was performed using an antisense primers annealing to the second exons of *BCAP29* gene. A fragment between the last intron and last exon of *DUS4L* was then amplified using the cDNA from above. (**B**) In RWPE-1, the correct sized band was observed in “+RT” (with reverse transcriptase); “-RT”, no reverse transcriptase. The RNA was pretreated with DNaseI. (**C**) Sanger sequencing validated the PCR fragment sequence. The sequence matches the fragment as demonstrated in UCSC Genome browser.

*DUS4L-BCAP29* is the type of chimeric transcript that combines the exons of adjacent genes, making them a candidate for cis-splicing between adjacent genes (cis-SAGe). This chimeric RNA also involves the second-to-last exon in *DUS4L* joining to the second exon in the *BCAP29*. As demonstrated in our previous study, this configuration is the most common type of cis-SAGe [9]. To prove its transcriptional read-through nature, we used a reverse primer annealing to the second exon of *BCAP29* as the RT primer, and detected the fragment of the primary transcript between the last intron and the last exon of *DUS4L* (Figure 5), suggesting that the transcript runs from *DUS4L* to *BCAP29*. To avoid DNA contamination, DNaseI treatment and “no reverse transcriptase” control (Supplementary Figure 4) were used as we described before [9, 24, 25].

## DISCUSSION

Traditionally, gene fusions resulting from chromosomal rearrangement are considered ideal biomarkers in clinical diagnostics. For instance, *PAX3-FOXO1* resulting from the t(2;13)(q35;q14) translocation [26] is detected in 55% of alveolar rhabdomyosarcoma (ARMS) [27], and it is used as a diagnostic aid in many pathology laboratories worldwide [28]. Fusion products can also be ideal therapeutic targets. Prominent examples include Gleevec targeting *BCR-ABL* in chronic myelogenous leukemia [5], and crizotinib targeting *EML4-ALK* in lung cancer [7].

However, recent research on intergenic splicing has demonstrated that trans-splicing and cis-SAGe are other mechanisms to generate chimeric RNAs [19], and that the chimeric RNAs can also exist in normal physiology [14–17]. For instance, *PAX3-FOXO1* fusion RNA, was detected transiently during muscle differentiation, but no evidence of t(2;13) was detected in the normal cells [20, 29]. The assumption that all fusion (RNA)s are cancer-fusions resulted in an explosion in the deposition of fusion genes into the Mitelman Database of Chromosome Aberrations and Gene Fusions in Cancer in the Cancer Genome Anatomy Project [30]. The number of entries reached 10,256 in June 2016, compared to only a few hundred entries several years ago. This is largely due to the wide application of RNA-Sequencing of cancer samples. However, the vast majority of studies did not include a sufficient number of normal, and non-cancer control samples. Rushing to translate these fusions into cancer biomarkers will result in a disastrous amount of false positives [31]. Previously, we found 13 fusions that are listed in the Mitleman database list as of April 2015, are also present in multiple non-cancer tissues [17], and *DUS4L-BCAP29* is one of them. Here, we provide further evidence to support that this fusion is not a cancer-specific event. In contrast, it is widely expressed in various tissues and cell types. Different from the traditional gene fusions produced via chromosomal rearrangement, it is a product of transcriptional read-through.

*DUS4L* encodes a dihydrouridine synthase 4 like protein. *BCAP29* encodes the B-cell receptor associated protein 29. Based on the sequence, the fusion creates an in-frame chimeric protein, which contains the TIM_phosphate_binding superfamily domain from DUS4L and BAP31 superfamily domain from *BCAP29*. The exact function of the fusion in normal and cancer cells is not clear. The loss-of-function system, as reported before [2], is not sufficient to support the function of the fusion, as the siRNA effect may be purely due to the silencing of wild type *DUS4L*. Further support for the idea comes from microarray analyses Interestingly, where we found that the most enriched GO term shared by wild type DUS4L silencing and the fusion silencing is negative regulators of apoptosis. Interestingly, the fusion may have some novel functions different from those of the parental genes, as evidenced by some unique GO terms only seen in siDUS4L-BCAP29 group. We are actively investigating the fusion functional mechanisms and their implications in normal physiology.

## MATERIALS AND METHODS

### Cell culture

The cell line GES-1 was cultured in RPMI1640 media containing 10% fetal bovine serum (Gibco^®^, Invitrogen, Carlsbad, CA), 100 units/mL penicillin, and 100 μg/mL streptomycin, at 37°C in a 5% CO2 humidified incubator. RWPE-1 cells were grown as previously described [26].

### RT-PCR and sanger sequencing

Total RNA was extracted with TRIzol reagent (Life Technologies, United States) and cDNA was generated by cDNA synthesis Kit (Bioline, United States), according to the manufacturer's instructions. Following RT-PCR and gel electrophoresis, purified DNA bands were sent for Sanger sequencing by Eton Bioscience INC (NC, United States).

### Real-time PCR

qRT-PCR was performed as described previously [9, 24, 32]. GAPDH was used as the endogenous control. The qPCR experiments were conducted with the ABI Step One Plus real time PCR system (Applied Biosystems, USA). The following primers are used for fusion and wild type RNAs. *DUS4L-BCAP29*-F: GCCAGTGC ACTATGATTCCA, *DUS4L-BCAP29*-R: GGAGGAATAA AAGGTAGGCAGAA; DUS4L-RT-F: TCCATGGAAG AACTGCTGAA, DUS4L-RT-R: AGAGTCCTCTTGCA ACCATCA. For the primer set to amplify fusion transcript, we designed the primers flanking the fusion junction. The forward primer anneals to the second-to-last exon of *DUS4L*, and the reverse primer anneals to the second exon of *BCAP29*. For primer sets specific for wild type parental genes, primer pairs annealing to only wild type but not fusion part were used.

### siRNA and transfection

siRNA was purchased from Invitrogen and siRNA transfection was carried out using Lipofectamine RNAiMAX (Life Technologies, United States) following the manufacturer's protocols. The siRNA sequence that was reported for silencing the *DUS4L-BCAP29* fusion transcript is 5′-AUUAAUACAGAUAUGUUUC- 3′ [22]. The siRNA sequence used against the *DUS4L* wide type transcript is 5′-AGGCAAACAUUGACAAAUA - 3′.

### *DUS4L–BCAP29* expression constructs

Human *DUS4L–BCAP29* sequence was amplified from GES-1 cell line by Reverse Transcription PCR, and cloned into mammalian expression vectors. The forward primer was 5′-CCGGAATTCCCACCATGAAGA GTGACTGCATGCAAACGACAATA- 3′ and the reverse primer was 5′-CGCTCGAGAAGCTTTCACAGTCTTT TCTTGTTGCCTCTTTCTAA- 3′. The cDNA was inserted into EcoRI/XhoI cloning sites of pCDNA3, or pQXCIH-CMV. Cells stably expressing the transgenes were selected with antibiotics, G418 (for pCDNA3.1), and hygromycin (for pQXCIH-CMV) respectively.

### Cell proliferation and migration assays

Cells were plated at proper density and then transfected with siRNAs, or expression plasmids and controls. The number of total cells in each well was counted after three days of transfection. Cell migration was measured by a wound-healing assay. Briefly, a wound was created by scraping the cells using a 10 μl plastic pipette tip, and the medium was replaced with fresh medium. Images were captured immediately after making the scratch, and again 12 hours later. Cell migration was qualitatively assessed by the size of the wounds at the end of the experiment, as previously described [25].

## SUPPLEMENTARY MATERIALS FIGURES



## Abbreviations

cis-SAGe: cis-Splicing of Adjacent Genes.
