# Supplementary material for: Recurrent fusion RNA DUS4L-BCAP29 in non-cancer human tissues and cells
Source: Oncotarget. 2017 Mar 17;8(19):31415–23. doi: 10.18632/oncotarget.16329 (PMC5458218; doi:10.18632/oncotarget.16329)
Supplement: Supplementary file 1 [file oncotarget-08-31415-s001.pdf]

Recurrent fusion RNA *DUS4L-BCAP29* in non-cancer human tissues and cells

Supplementary Materials

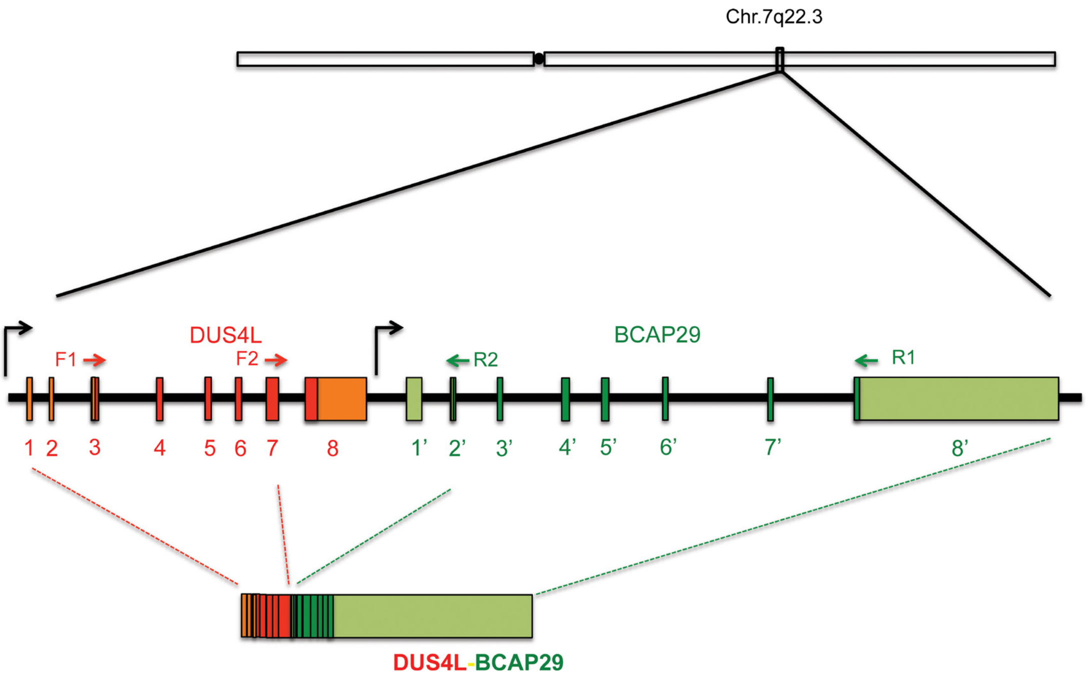

Supplementary Figure 1: Scheme of the fusion and parental genes. Colored blocks represent exons, whereas lines represent introns and the intergenic region. Darker color indicates the protein-coding region, and fainter color indicates the untranslated region. F1 and R1 primers were used to amplify the full-length coding region. F2 and R2 primers were used for detecting the fusion in qRT-PCR and RT-PCR.

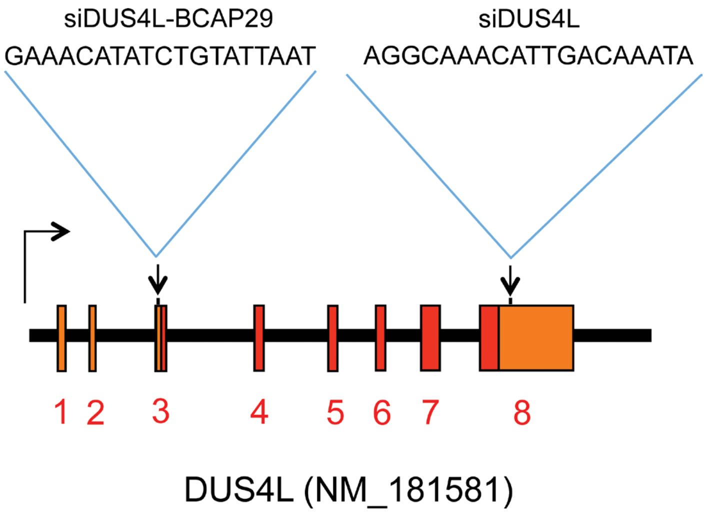

Supplementary Figure 2: Targeting positions of siDUS4L-BCAP29 and siDUS4L. Locations of the targeting sequence for siDUS4L-BCAP29 and siDUS4L are marked. siDUS4L-BCAP29 targets both wild type *DUS4L* and the fusion, whereas siDUS4L only targets wild type *DUS4L*.

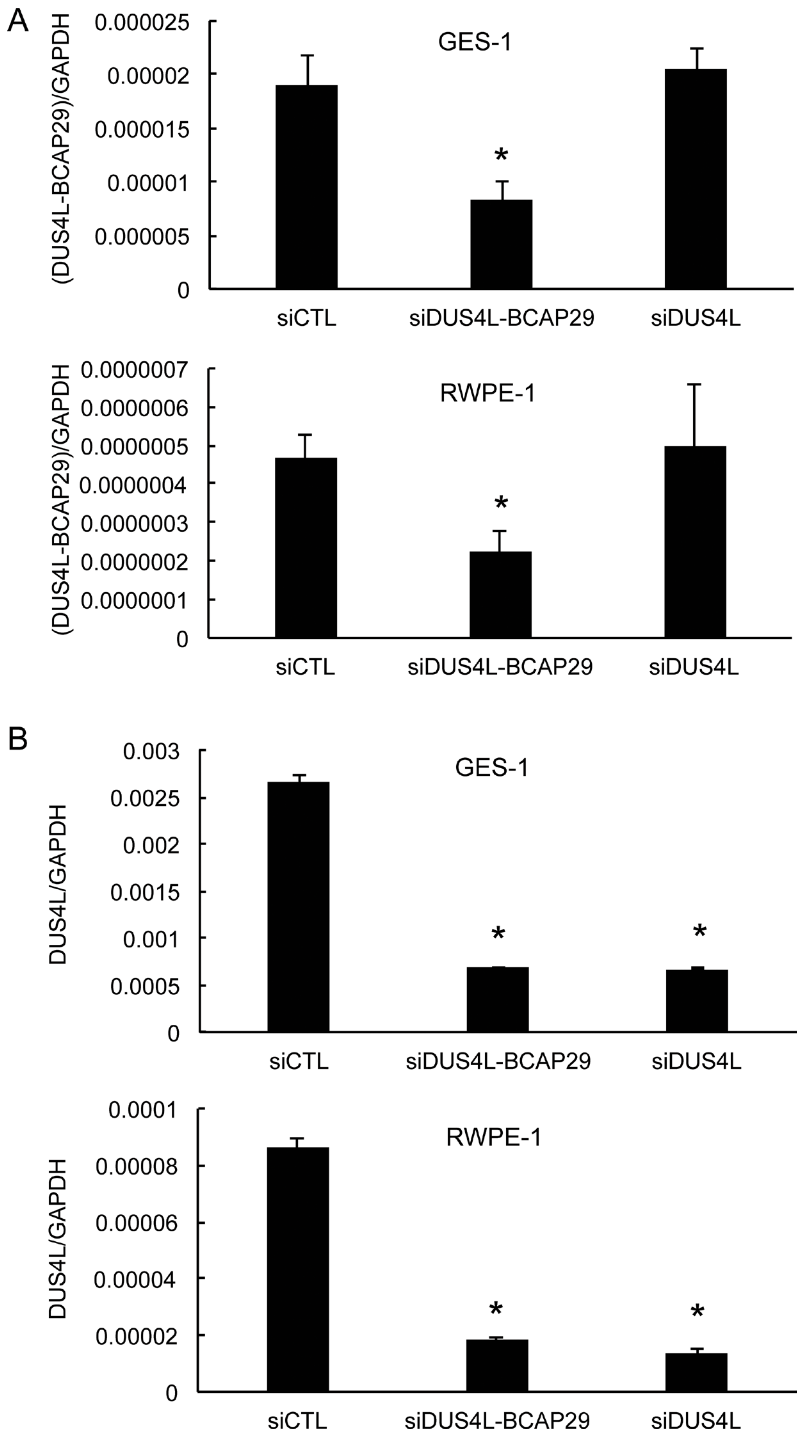

Supplementary Figure 3: Effect of the two siRNAs. (**A**) Effects of the two siRNAs on the fusion RNA in RWPE-1 and GES-1. As expected, only siDUS4L-BCAP29 silenced the fusion. (**B**) Effects of the two siRNAs on wild type *DUS4L* in RWPE-1 and GES-1. As expected, both silenced the wild type transcript to a similar extent. $*p < 0.05$.

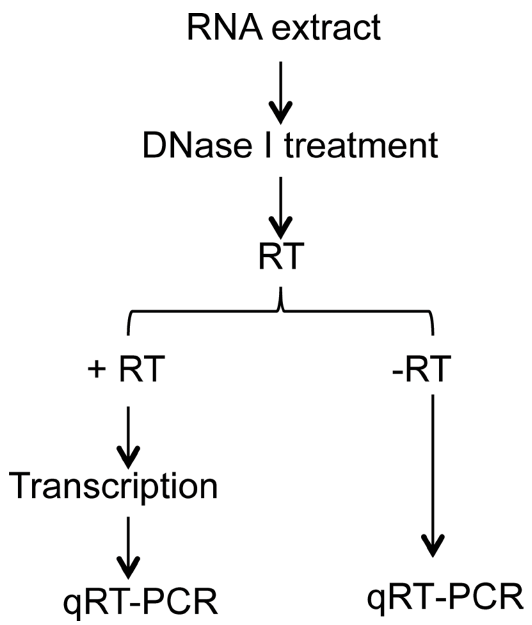

Supplementary Figure 4: The workflow of the RT-PCR to detect primary transcript running from *DUS4L to BCAP29*.

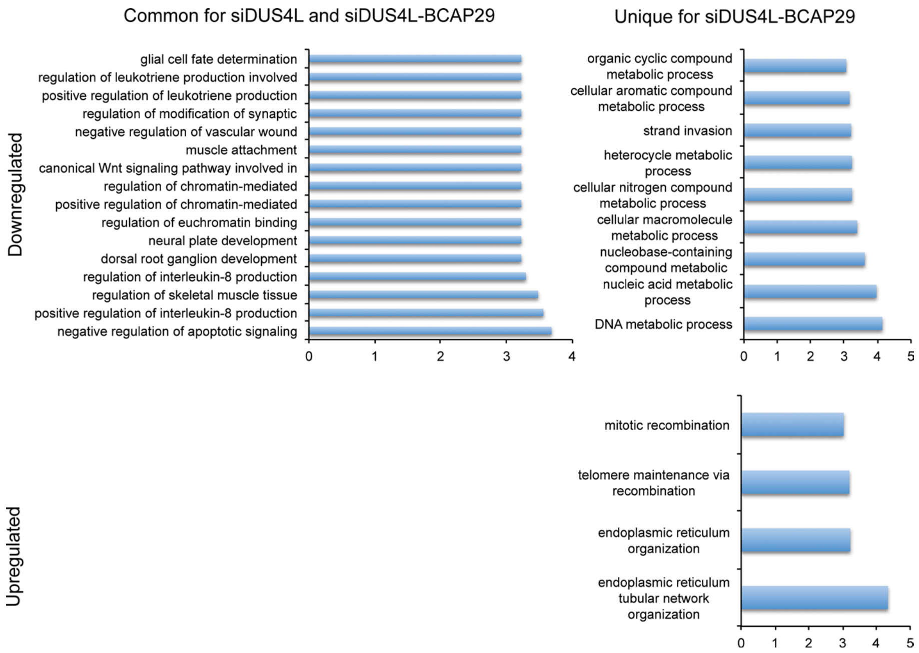

Supplementary Figure 5: Gene ontology terms enriched in siDUS4L-BCAP29 and siDUS4L compared with control siCTL. Plotted are statistical significances (-Log10(p-value)) of each term. On the left, are GO terms enriched in both. On the right are terms enriched only in siDUS4L-BCAP29.
